# Cytokine dysregulation despite immunoglobulin replacement therapy in common variable immunodeficiency (CVID)

**DOI:** 10.3389/fimmu.2023.1257398

**Published:** 2023-09-28

**Authors:** Remo Poto, Antonio Pecoraro, Anne Lise Ferrara, Alessandra Punziano, Gianluca Lagnese, Carla Messuri, Stefania Loffredo, Giuseppe Spadaro, Gilda Varricchi

**Affiliations:** ^1^ Department of Translational Medical Sciences, University of Naples Federico II, Naples, Italy; ^2^ World Allergy Organization (WAO), Center of Excellence (CoE), Naples, Italy; ^3^ Unità Operativa (UO) Medicina Trasfusionale, Azienda Sanitaria Territoriale, Ascoli Piceno, Italy; ^4^ Center for Basic and Clinical Immunology Research (CISI), University of Naples Federico II, Naples, Italy; ^5^ Institute of Experimental Endocrinology and Oncology, National Research Council (CNR), Naples, Italy

**Keywords:** common variable immunodeficiency, CVID, lipopolysaccharide, IL-1, TNF-α, IL-6, IL-10

## Abstract

**Introduction:**

Common variable immunodeficiency (CVID) is the most prevalent symptomatic primary immunodeficiency. CVID is a heterogeneous disorder with a presumed multifactorial etiology. Intravenous or subcutaneous immunoglobulin replacement therapy (IgRT) can prevent severe infections but not underlying immune dysregulation.

**Methods:**

In this study, we evaluated the serum concentrations of proinflammatory (TNF-α, IL-1β, IL-6) and immunoregulatory cytokines (IL-10), as well as lipopolysaccharide (LPS) and soluble CD14 (sCD14) in CVID individuals with infectious only (INF-CVID), and those with additional systemic autoimmune and inflammatory disorders (NIC-CVID), and healthy donors (HD).

**Results:**

Our results showed increased serum concentrations of TNF-α, IL-1β, IL-6, and IL-10 in both INF-CVID and NIC-CVID subjects compared to HD. However, elevations of TNF-α, IL-1β, IL-6, and IL-10 were significantly more marked in NIC-CVID than INF-CVID. Additionally, LPS concentrations were increased only in NIC-CVID but not in INF-CVID compared to HD. Circulating levels of sCD14 were significantly increased in NIC-CVID compared to both INF-CVID and HD.

**Discussion:**

These findings indicate persistent cytokine dysregulation despite IgRT in individuals with CVID. Moreover, the circulating cytokine profile reveals the heterogeneity of immune dysregulation in different subgroups of CVID subjects.

## Introduction

Common variable immunodeficiency (CVID) is the most prevalent symptomatic primary immunodeficiency characterized by low immunoglobulin (Ig) levels (IgG and/or IgM) and impaired production of specific antibodies in response to vaccinations (1, 2). CVID is a heterogeneous disorder (3, 4) with a presumed multifactorial etiology and an estimated heritability of approximately 20% (2, 5–7).

Intravenous or subcutaneous immunoglobulin replacement therapy (IgRT) is effective in preventing severe infections and improving survival in CVID subjects (1, 3, 8). However, despite optimal IgRT, a significant percentage (30% to 50%) of these patients still experience complications related to immune dysregulation (3, 9–11). These complications include lymphoproliferative and autoimmune diseases, cytopenias, enteropathy, chronic lung diseases, and cancer (9, 12–16). The mechanisms underlying these heterogeneous manifestations of immune dysregulation remain unclear (17–19). Recent evidence suggests that gut microbiome alterations may be a driver of immune dysregulation in CVID subjects (20, 21).

Early identification of predictive biomarkers and risk factors for the development of immune dysregulation in CVID is crucial to improve the clinical care of these patients. Previous studies have suggested several potential risk factors, including low CD4^+^ T cells (22), increased CD21^low^ B cells (23, 24), IgA deficiency (22, 24) and gut dysbiosis (21). More recently, the measurement of circulating cytokines and chemokines is gaining ground in identifying CVID immunophenotypes with different clinical manifestations (12, 13, 25–27). However, previous studies have reported conflicting results about serum cytokines in CVID subjects compared to healthy donors. For instance, serum concentrations of IL-10 have been found to be both increased (28, 29) or decreased (30) in CVID subjects compared to controls. These apparent discrepancies could be attributed, at least in part, to the selection of CVID individuals with different immunophenotypes.

In this study, we have evaluated the cytokine profile and biomarkers of systemic inflammation in CVID individuals with a clinical history of either infections only (INF-CVID) or autoimmune disease, GLID, lymphoproliferation, splenomegaly, bronchiectasis, and/or enteropathy (NIC-CVID) undergoing IgRT compared to healthy donors.

## Materials and methods

### Subjects

Forty-six Caucasian individuals with CVID were recruited from the outpatient clinic of the Division of Allergy and Clinical Immunology, University of Naples Federico II (Naples, Italy). Twenty-three healthy volunteers (HD) were enrolled as age and gender-matched controls. Patients were eligible for enrollment in the study if they had a CVID diagnosis based on the 2019 ESID criteria (31). Exclusion criteria for both healthy donors and CVID subjects were active bacterial or viral infections at the time of blood collection. There were no familiar cases of CVID. All patients were receiving chronic IgRT and were not undergoing immunosuppressive therapy. From the medical files of CVID subjects, we recorded serum Ig levels at diagnosis, T cell subsets, monocytes, B cells, clinical history of recurrent infections, chronic diarrhea, bronchiectasis, autoimmune diseases, polyclonal lymphoproliferation (splenomegaly, lymphadenopathy, and granulomatous disease), and malignancies. This study was approved by the institutional Ethics Committee of the University of Naples Federico II (Prot. 198/18), and informed consent was obtained from all participants prior to blood collection according to recommendations from the Declaration of Helsinki.

### IgG, IgA, and IgM measurements

Serum samples obtained from venous blood were aliquoted and stored at − 80°C until testing. IgG, IgA, and IgM were measured by nephelometry employing the Behring BN™ II System (Siemens Healthcare Diagnostics Ltd, Erlangen, Germany) (32). To evaluate the precision of immunoglobulin measurement, the coefficient of variation (CV) was determined according to the Clinical and Laboratory Standards Institute (CLSI) EP05-A3 guidelines (33). The intra-assay CVs ranged from 1.8% to 3.6%. The linearity of the IgG measurement (coefficient of determination: R^2^) was assessed according to the CLSI EP06-A guidelines (34). The R^2^ values ranged from 0.97 to 0.99.

### Cytokine, soluble CD14, and LPS measurements

Serum concentrations of TNF-α (Quantikine HS ELISA HSTA00E), IL-1β (Quantikine HS ELISA HSLB00D), IL-6 (Quantikine HS ELISA HS600C), and IL-10 (Quantikine HS HS100C) were measured in duplicate using ELISA assays from R&D Systems (Bio-Techne). The concentration of soluble CD14 (sCD14) was measured using the Quantikine ELISA DC140 kit from R&D Systems (Bio-Techne). The concentration of lipopolysaccharide (LPS) was also measured using an ELISA kit from Abbexa (Cambridge, UK). All reagents and chemicals used in these experiments were molecular biology grade to reduce endotoxin contamination. Disposable pipets, tubes, and plates were used in all experiments.

### Statistical analysis

Statistical analysis was performed using GraphPad Prism 9 software (GraphPad Software, La Jolla, CA, USA). Data are presented as mean ± standard error of the mean (SEM). Group comparisons were made using Student’s t-test or Mann–Whitney U test, based on the parametric or nonparametric distribution of the continuous variables. Multiple comparisons were assessed using one-way analysis of variance (ANOVA) followed by Tukey’s *post hoc* test. Correlation analyses were conducted using Pearson’s correlation method. A *p* value less than 0.05 was considered statistically significant.

## Results

### Demographic and clinical characteristics of CVID subjects and healthy donors

CVID subjects were categorized as INF-CVID if they had a clinical history of recurrent infections only. NIC-CVID included patients with recurrent infections as well as one or more of the following non-infectious complications: enteropathy, lymphoproliferation, autoimmune disease, cytopenia, splenomegaly, granulomatous lymphocytic interstitial lung disease (GILD) or bronchiectasis. All CVID subjects were on IgRT at the time of sample collection.

**Table 1** presents the demographic and clinical characteristics of healthy donors (HD), while **Tables 2**, **3** provide the characteristics of 23 subjects with INF-CVID and 23 subjects with NIC-CVID, respectively. The median age was 44.5 ± 2.5 years for INF-CVID, 41.3 ± 2.5 years for NIC-CVID, and 39.6 ± 2.2 years for HD. Among the INF-CVID patients, eleven (47.8%) were female and among NIC-CVID ten (43.4%) were female.

**Table 1:** Demographic and clinical characteristics of healthy donors (HD).

HD	Gender (M/F)	Age (years)	IgG (mg/dL)	IgA (mg/dL)	IgM (mg/dL)
1	F	23	1030	91	68
2	M	40	1050	134	93
3	M	51	1130	253	199
4	F	24	982	269	130
5	M	30	1230	167	82
6	F	48	1370	144	121
7	M	54	1070	124	101
8	M	33	1180	106	48
9	F	35	1160	131	86
10	F	37	774	246	201
11	M	42	1420	186	131
12	F	45	955	148	85
13	F	31	990	189	73
14	M	29	838	261	164
15	M	45	859	281	173
16	F	25	907	212	135
17	M	50	850	205	138
18	F	34	824	117	87
19	M	41	1070	276	172
20	F	28	1350	216	149
21	M	49	890	194	130
22	F	59	1060	224	135
23	M	58	775	201	102
Mean ± SEM		39.61 ± 2.28	1033 ± 39.25	190.2 ± 12.12	121.9 ± 8.72

**Table 2:** Demographic and clinical characteristics of CVID subjects with recurrent infections (INF-CVID).

INF-CVID subjects	Gender (M/F)	Age (years)	IgG at diagnosis (mg/dL)	IgA at diagnosis (mg/dL)	IgM at diagnosis (mg/dL)	IgG through level (mg/dL)
1	M	40	7	7	0	643
2	F	22	360	8	31	1070
3	M	46	41	5	27	616
4	F	49	103	5	0	484
5	F	48	312	28	29	733
6	F	50	50	5	30	1160
7	M	36	167	5	11	1030
8	F	49	134	0	17	870
9	M	61	104	10	16	625
10	F	54	175	9	56	940
11	M	60	346	24	43	1005
12	F	55	15	0	10	650
13	M	48	84	0	0	875
14	F	62	5	0	0	740
15	M	43	168	0	20	573
16	M	51	96	21	0	486
17	F	29	250	0	5	525
18	M	23	116	10	0	638
19	M	39	83	0	0	1080
20	F	27	206	5	21	1100
21	M	51	412	7	10	636
22	F	25	102	0	0	1078
23	M	57	114	0	5	910
Mean ± SEM		44.57 ± 2.58	150.0 ± 24.12	6.47 ± 1.66	14.39 ± 3.27	802.9 ± 46.28

**Table 3:** Demographic and clinical characteristics of CVID subjects with addition of a variety of immune disorders (NIC-CVID).

NIC-CVID subjects	Gender (M/F)	Age (years)	IgG at diagnosis (mg/dL)	IgA at diagnosis (mg/dL)	IgM at diagnosis (mg/dL)	IgG through level (mg/dL)
1	F	42	5	0	0	785
2	F	24	272	0	0	1080
3	M	53	109	0	0	760
4	F	25	167	0	0	785
5	M	54	90	17	37	767
6	M	30	23	0	15	1160
7	M	45	28	5	0	755
8	M	47	260	5	0	476
9	F	38	275	5	0	649
10	F	36	112	5	0	1100
11	M	33	85	5	0	1010
12	M	53	300	50	20	869
13	M	47	164	42	0	619
14	M	29	209	0	12	941
15	M	23	433	35	72	1010
16	F	50	186	0	0	648
17	F	41	169	5	0	473
18	M	21	404	58	39	471
19	F	59	96	14	19	510
20	F	61	310	16	21	1090
21	M	55	38	5	29	980
22	F	48	115	15	0	1150
23	M	36	251	0	12	690
Mean ± SEM		41.30 ± 2.53	178.3 ± 24.85	12.26 ± 3.59	12.00 ± 3.80	816.4 ± 47.54

### Serum concentrations of IgG, IgA, and IgM in INF-CVID, NIC-CVID, and healthy donors

**Figure 1:**
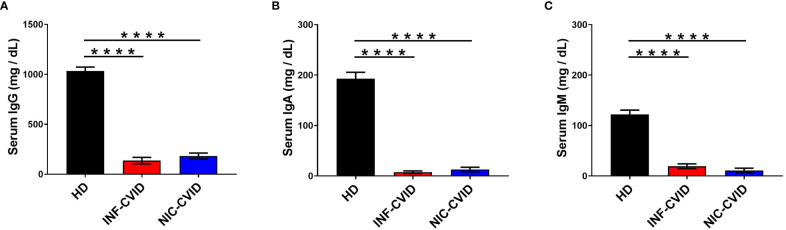
Serum concentrations of IgG **(A)**, IgA **(B)**, and IgM **(C)** in healthy donors (HD), CVID subjects with recurrent infections only (INF-CVID) or with addition of a variety of immune disorders (NIC-CVID). **** *p* < 0.0001.

We evaluated the serum concentrations of IgG, IgA, and IgM in subjects with INF-CVID or NIC-CVID compared to HD. **Figure 1A** shows that both INF-CVID and NIC-CVID had significantly lower IgG serum concentrations than HD (*p* < 0.0001). N difference was found between the two groups of CVID. Similarly, serum concentrations of both IgA (**Figure 1B**) and IgM (**Figure 1C**) were markedly lower in both CVID groups compared to HD (*p* < 0.0001).

### Serum concentrations of cytokines in INF-CVID, NIC-CVID, and healthy donors

**Figure 2:**
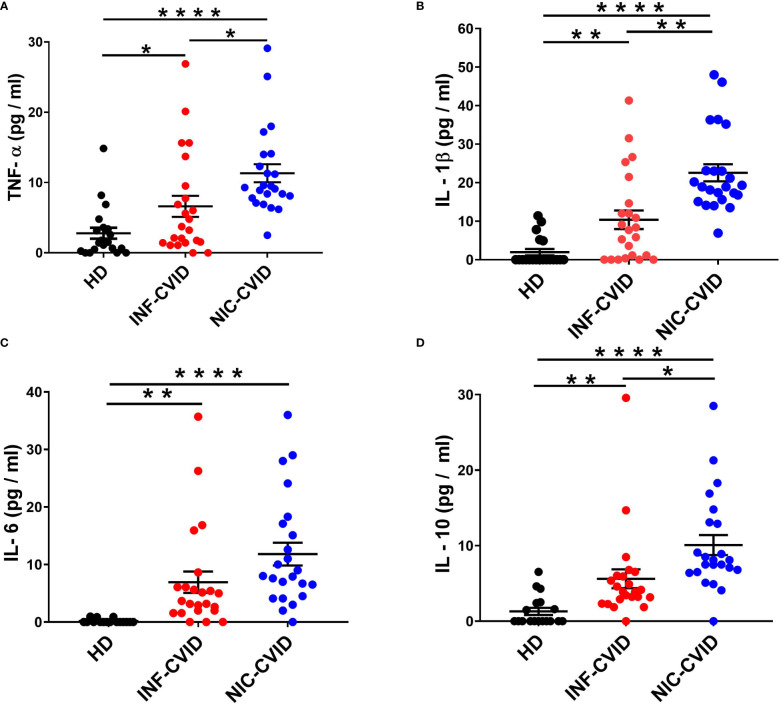
Serum concentrations of TNF-α **(A)**, IL-1β **(B)**, IL-6 **(C)**, and IL-10 **(D)** in healthy donors (HD), CVID subjects with recurrent infections only (INF-CVID) or with addition of a variety of immune disorders (NIC-CVID). * *p* < 0.05; ** *p* < 0.01; **** *p* < 0.0001.

To investigate the mechanisms underlying chronic immune dysregulation in CVID, we measured serum levels of different cytokines in subjects with INF-CVID and NIC-CVID compared to HD. We found a significant elevation of TNF-α in both INF-CVID and NIC-CVID compared to HD (**Figure 2A**). Elevation of serum TNF-α was more marked in NIC-CVID compared to INF-CVID. Similarly, serum concentration of IL-1β were increased in both INF-CVID and NIC-CVID compared to HD (**Figure 2B**). The proinflammatory cytokine IL-6 was significantly increased in both INF-CVID and NIC-CVID, compared to HD (**Figure 2C**). The immunomodulatory cytokine IL-10 was significantly increased in both INF-CVID and NIC-CVID compared to HD (**Figure 2D**). Serum concentrations of IL-10 were more markedly increased in NIC-CVID compared to NF-CVID.

### Serum concentrations of LPS and sCD14 in INF-CVID, NIC-CVID, and healthy donors

**Figure 3:**
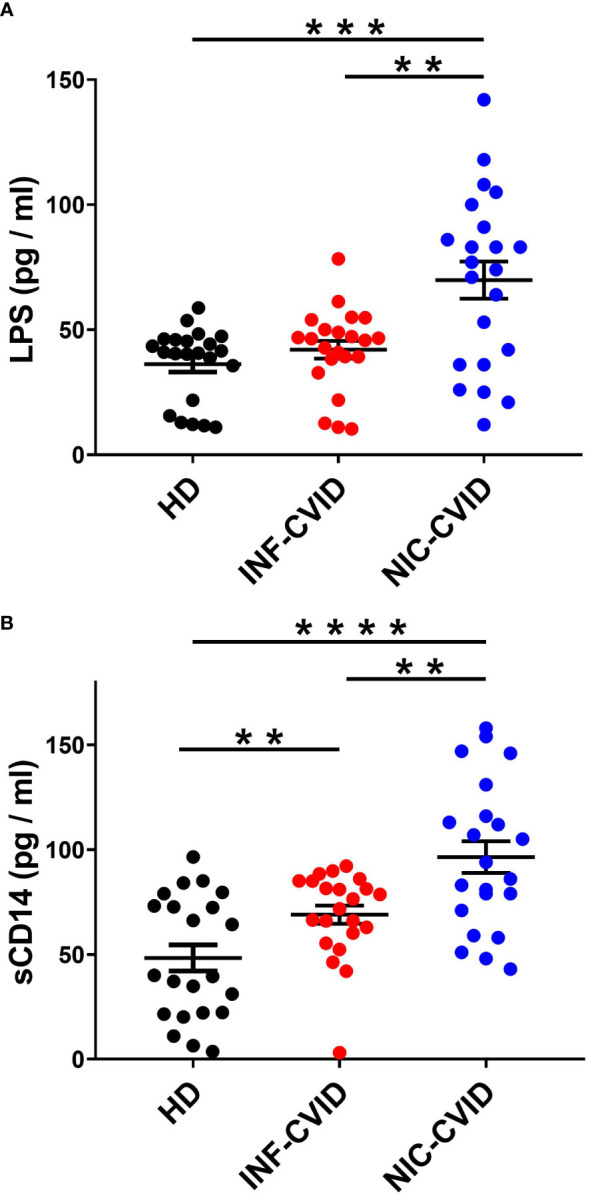
Serum concentrations of LPS **(A)** and sCD14 **(B)** in healthy donors (HD), CVID with recurrent infections only (INF-CVID) or with addition of a variety of immune disorders (NIC-CVID). ** *p* < 0.01; *** *p* < 0.001; **** *p* < 0.0001.

LPS is the major component of the outer membrane of Gram-negative bacteria (35, 36). LPS activates the multimeric receptor composed of Toll-like receptor-4 (TLR4), CD14, and MD2 on the membrane of several immune cells (e.g., macrophages, monocytes, neutrophils) (35–38). Circulating concentration of LPS is considered a biomarker of gut microbial translocation, a process facilitated by immune deficiency that may drive inflammation *via* endotoxemia (20, 39). Serum concentrations of LPS were not elevated in INF-CVID compared to HD (**Figure 3A**). By contrast, NIC-CVID subjects showed increased levels of LPS compared to both INF-CVID and HD (**Figure 3A**).

CD14 is a glycosylphosphatidylinositol (GPI)-anchored receptor which serves as a co-receptor for Toll-like receptor (TLR)-4 at the cell surface or can be secreted in a soluble form (sCD14) (40, 41). To assess the degree of systemic immune activation in CVID, we analyzed the serum levels of sCD14, a recognized marker of monocyte activation (42). Serum concentrations of sCD14 were increased in INF-CVID compared to HD. Moreover, sCD14 levels were significantly increased in NIC-CVID compared to both INF-CVID and HD (**Figure 3B**).

### Correlations between cytokines, LPS, sCD14, and IgG concentrations

In our study, we examined the correlations between cytokine (TNF-α, IL-6, IL-1β, IL-10) levels and IgG concentrations after IgRT in patients with INF-CVID (**Supplementary Figure 1**: **sFigure1**) and NIC-CVID (**Supplementary Figure 2**: **sFigure 2**). No significant correlations were observed in both groups of CVID patients. Previous studies have reported an inverse correlation between endotoxemia and serum IgG levels in CVID patients (43). However, in our study, we did not find any correlation between LPS and sCD14 levels with IgG concentrations (**Supplementary Figures 3**, **4**: **sFigures 3**, **4**). No correlations were also found between cytokine levels and sCD14 concentrations in patients with INF-CVID (**Supplementary Figure 5**: **sFigure 5**) and NIC-CVID (**Supplementary Figure 6**: **sFigure 6**). Similarly, no correlations were found between cytokines and LPS in patients with INF-CVID (**Supplementary Figure 7**: **sFigure 7**) and NIC-CVID (**Supplementary Figure 8**: **sFigure 8**). No correlations were also found between sCD14 and LPS concentrations in both patients with INF-CVID and NIC-CVID (**Supplementary Figure 9**: **sFigure 9**).

### Correlations between the different cytokines in the single subjects with INF-CVID and NIC-CVID

We have also examined the correlations between the different cytokines in the single subjects with both INF-CVID (**Supplementary Figure 10**: **sFigure 10**) and NIC-CVID (**Supplementary Figure 11**: **sFigure 11**). No correlations were found between the different cytokines with the exception of the significant correlation between IL-10 and IL-1β in INF-CVID subjects (*p* < 0.001).

## Discussion

The results of this observational study demonstrate that individuals with different CVID phenotypes exhibit varying degrees of systemic cytokine dysregulation in peripheral blood. In particular, low-grade inflammation is more evident in subjects with NIC-CVID compared to INF-CVID. This condition of systemic inflammation, characterized by elevated levels of proinflammatory (i.e., TNF-α, IL-1β, IL-6) and immunoregulatory cytokines (i.e., IL-10) persists in CVID subjects despite receiving adequate IgG replacement therapy. Interestingly, we did not find significant correlations between serum concentrations of IgG and several inflammatory markers.

CVID is the most prevalent symptomatic primary immunodeficiency (44, 45). Although genetic etiologies have been identified in approximately 20% of CVID cases (2, 5–7), the pathobiological mechanisms underlying the inflammatory manifestations remain largely unknown (17, 18). Moreover, CVID is a highly heterogeneous condition (3, 4) with different immunological phenotypes characterized by various complications (12, 21, 26, 27, 46). For instance, serum and sputum cytokine levels are markedly increased in CVID subjects with chronic respiratory symptoms and airway disease compared to those with normal airways (26). Similarly, CVID subjects with autoimmune cytopenias (AIC) and lymphadenopathy have elevated levels of LPS and exhibit hyperplastic but inefficient germinal center responses compared to CVID individuals without AIC (46). Previous studies by Jorgensen and collaborators demonstrated that plasma levels of LPS, IL-6, and TNF-α were increased in CVID subjects with dysimmune complications (e.g., splenomegaly, lymphadenopathy, autoimmunity, enteropathy, granulomatous-lymphocytic interstitial lung disease [GLILD]) compared to CVID without dysimmune complications (27). Our findings confirm and extend the previous observations, showing that serum levels of inflammatory markers (LPS, sCD14) and cytokines (TNF-α, IL-1β, IL-6) are increased in NIC-CVID subjects compared to INF-CVID. Moreover, we found that circulating levels of the immunoregulatory cytokine IL-10 were also increased in both NIC-CVID and INF-CVID subjects.

LPS is a glycoprotein released by Gram-negative bacteria, which represent the vast majority of gut microbiota (47). Circulating LPS is considered a parameter of increased intestinal permeability and/or microbial translocation of Gram-negative bacteria (48). LPS activates the innate immune system by interacting with Toll-like receptor 4, an important mediator of innate immunity (35, 49). Previous studies investigating the circulating levels of LPS in individuals with CVID have yielded conflicting results, likely due to the inherent heterogeneity among CVID subjects (12, 27, 43, 46, 50, 51). Furthermore, the assays employed in these studies are known to exhibit high variability, making it challenging to compare findings obtained using different experimental methods (e.g., serum *vs*. plasma). Finally, it is known that sCD14 can directly bind to LPS (52, 53) and therefore can interfere with the measurement of circulating endotoxin (54). Initial studies reported that circulating levels of LPS were not increased in unselected CVID subjects (50, 51). By contrast, Perreau and coworkers found that plasma levels of LPS were markedly increased in CVID subjects before IVIG therapy (43). Also recent studies measuring circulating LPS in different CVID subgroups compared to HD have been controversial (12, 27, 46). Two studies highlighted significant differences between CVID subgroups showing that circulating levels of LPS are increased in CVID with inflammatory complications (27) and with autoimmunity (46) compared to uncomplicated CVID. By contrast, another recent study reported no differences in plasma LPS in patients with uncomplicated and complicated CVID and controls (12). In our study, we found that LPS levels are more markedly increased in NIC-CVID compared to INF-CVID.

Jorgensen and collaborators elegantly demonstrated that increased plasma levels of LPS in CVID subjects were associated with systemic inflammation and enhanced macrophage and T-cell activation (27). Interestingly, LPS concentration correlated with gut microbial dysbiosis in stool samples in CVID. More recently, it has been shown that gut mucosa of CVID subjects has altered response to LPS and viruses (55). Further studies are required to investigate the true extent of endotoxemia and its association with systemic and/or local (i.e., gastrointestinal) immune dysregulation in different immunophenotypes of CVID.

It should be pointed out that different commercial preparations of IVIG contain a broad spectrum of anti-carbohydrate antibodies, including bacterial glycans (56). In particular, the bacterial constituents containing these glycans include the cell wall component LPS. These findings suggest that administration of IVIG might interfere with the measurement of circulating LPS in CVID subjects. Indeed, a study examining the presence of endotoxin in CVID subjects before and after IVIG therapy reported a decrease in endotoxin levels following immunoglobulin treatment (43). This decrease was attributed to the reduction of bacterial translocation caused by IVIG in CVID subjects.

Soluble CD14 (sCD14) is a circulating marker of monocyte activation (40). Previous reports on circulating levels of sCD14 in CVID have shown conflicting results (12, 27, 50, 51, 57, 58). Initial studies reported increased serum concentrations of sCD14 in unselected CVID subjects (50, 51), while others found no significant difference compared to controls (58). Recently, two studies reported that plasma levels of sCD14 were increased in CVID, particularly in those with complications (12, 27). Our results are consistent with the latter findings, showing that sCD14 concentrations are more markedly increased in NIC-CVID compared to INF-CVID.

LPS activates the TLR4 on human macrophages inducing the release of several proinflammatory cytokines, including TNF-α, IL-1β, and IL-6 (36, 59). Circulating levels of TNF-α were found to be increased in CVID compared to controls by several investigators (12, 13, 26, 27, 29). Our results showing that circulating levels of TNF-α are more markedly increased in NIC-CVID compared to INF-CVID, are consistent with more recent findings (12, 26). IL-1β is a classical proinflammatory cytokine mainly produced by human macrophages and neutrophils (60, 61). To the best of our knowledge, there is only one published study that has investigated the serum concentration of IL-1β in CVID subjects (26). Schnell and collaborators reported that serum concentrations of IL-1β were increased in CVID compared to controls. Moreover, circulating and sputum levels of IL-1β were more markedly increased in CVID with abnormal airways compared to normal airways (26). Our results extend the previous findings showing that serum concentrations of IL-1β are more markedly increased in NIC-CVID compared to INF-CVID.

IL-6 is a proinflammatory cytokine mainly produced by human macrophages (62, 63). Several groups of investigators reported increased circulating levels of IL-6 in CVID (12, 13, 26, 27). In particular, elevated levels of IL-6 were found in CVID with abnormal airways (26) or with complications (12) compared to controls. Our study extends the latter observations showing that serum levels of IL-6 are particularly increased in NIC-CVID compared to INF-CVID.

IL-10 is a pleiotropic cytokine that stimulates several immune cells and exerts immunosuppressive effects on myeloid cells (64). IL-10 is a powerful immune mediator with complicated, even opposite, biological effects. Several investigators reported increased circulating IL-10 levels in CVID subjects (12, 13, 25, 28, 29). In particular, in a study performed in two large cohorts, serum concentrations of IL-10 were increased in CVID with immune dysregulation compared to CVID with infections only (13). In another study, circulating levels of IL-10 were found to be increased in CVID with non-infectious complications compared to CVID with infections only (12). Our results extend the previous observations showing more pronounced elevation of IL-10 in NIC-CVID compared to INF-CVID. These results are apparently surprising. IL-10 is constitutively expressed by regulatory T cells (Treg cells) (65, 66). In CVID subjects, a low number of Treg cells has been associated with autoimmune manifestations (67–69). Moreover, reduced IL-10 production by Treg cells has been found in CVID subjects (70). However, it is possible that increased circulating levels of IL-10 in CVID are influenced by IVIG therapy, which selectively activates Treg cells (71). This hypothesis is supported by the observation that IVIG administration in mice increased IL-10 levels compared to untreated mice (72). Moreover, polyclonal immunoglobulins induced IL-10 from mouse macrophages *in vitro* (73).

The alteration of circulating cytokines in CVID subjects, as reported by us and other investigators (12, 13, 25–27, 29), may be attributed to intrinsic immunologic changes or induced by exogenous immunoglobulin administration. The first hypothesis is indirectly supported by a wide spectrum of inflammatory and immunologic alterations in different phenotypes of CVID patients (25). The second hypothesis finds support in experimental mouse models. For instance, it has been demonstrated that IVIG administration in mice increased the circulating levels of several cytokines (e.g., IL-10, IL-33) (72, 74). Interestingly, IVIG potentiated LPS-induced IL-10 release from mouse bone marrow-derived macrophages (73). This observation raises the possibility that increased levels of circulating LPS in CVID subjects could modulate the serum concentrations of certain cytokines. Similarly, there is the possibility that IVIG might induce significant alterations of innate and adaptive compartments in peripheral blood of CVID subjects. For instance, it has been proposed that IVIG could decrease the number of circulating inflammatory monocytes (75).

CVID patients with autoimmune and/or inflammatory complications have a higher mortality rate compared to patients with infections only (3, 9, 76, 77). In addition, there is compelling evidence that CVID subjects are associated with increased prevalence of lymphoproliferative disorders and other malignancies (9, 10, 14, 15, 78–81). In particular, an increased prevalence of malignant lymphoma (82) and gastric cancer (15) has been reported in CVID compared to the general population. In the past, this phenomenon was simplistically attributed to defects in B and/or T cell levels or functions (14, 22, 83, 84). More recently, it has been recognized that several risk factors can contribute to malignant transformation in primary immunodeficiencies (78). Among these factors, low-grade inflammation is emerging as one of the hallmarks of cancer (78, 85–87). The present and previous studies have demonstrated the presence of increased concentrations of at least two biomarkers (e.g., cytokines, LPS) of low-grade inflammation in peripheral blood of CVID subjects (12, 13, 25–27, 29, 43, 46). The first biomarker is represented by an increase of circulating proinflammatory cytokines (e.g., IL-1β, TNF-α, IL-6) demonstrated in previous (12, 13, 26, 27, 29) and in our study. These cytokines are involved in tumor initiation and growth (61, 62, 88). Another relevant biomarker is represented by an increase in LPS concentrations, particularly in subjects with complicated CVID (17, 27, 43). Recent evidence has demonstrated that LPS can favor the onset of cancer (89, 90) and the formation of metastasis (91, 92). These observations suggest that persistent elevation of proinflammatory cytokines and LPS concentrations, despite IgRT, could contribute to chronic inflammation, favoring the onset of solid and hematologic cancers in CVID subjects.

Our study has several limitations that should be pointed out. First, our study is limited by the small sample size of subgroups of CVID subjects and healthy donors. Multicenter studies examining CVID patients with different complications (e.g., lymphoproliferative, autoimmune and gastrointestinal diseases, cancer) will be necessary to identify specific circulating biomarkers of immune dysregulation (e.g., cytokines, chemokines). In addition, the small sample sizes of INF-CVID and NIC-CVID subjects from a single center do not allow for meaningful statistical correlations with clinical features and phases of clinical activity. Moreover, in this study we measured a limited number of circulating proinflammatory and immunomodulatory cytokines. Additional investigations are required to evaluate a wider spectrum of cytokines, including epithelium-derived alarmins (e.g., TSLP, IL-33, IL-25) (93–96), and chemokines (25, 97), presumably involved in immune dysregulation in patients with different CVID phenotypes. Moreover, the enrolled individuals with different phenotypes of CVID lacked a molecular diagnosis. Finally, multiple LPS-binding proteins have been found in human serum. Among these, LPS-binding protein (LBP) and bactericidial/permeability increasing protein (BPI) both bind the Lipid A component of LPS (98, 99). In this study, we did not evaluate the concentrations of LBP, BPI, plasma phospholipid transfer protein (PLTP) (100, 101) and LPS-neutralizing capacity (LPS-NC) (102), which could interfere with LPS measurement.

Despite these limitations, our findings expand previous results (25, 28, 29), indicating that several biomarkers of systemic inflammation persist in CVID patients, particularly in those with inflammatory and autoimmune complications (12, 13, 17, 26, 27), despite receiving IgRT. Further efforts appear necessary to understand the possible pathophysiological implications, particularly in relation to cancer development, of cytokine dysregulation in CVID subjects with different phenotypes.

## Data availability statement

The raw data supporting the conclusions of this article will be made available by the authors, without undue reservation.

## Ethics statement

The studies involving humans were approved by Ethics Committee of the University of Naples Federico II (Prot. 198/18). The studies were conducted in accordance with the local legislation and institutional requirements. The participants provided their written informed consent to participate in this study.

## Author contributions

RP: Conceptualization, Data curation, Formal Analysis, Investigation, Methodology, Validation, Writing – original draft, Writing – review & editing. AnP: Conceptualization, Data curation, Formal Analysis, Investigation, Writing – original draft, Writing – review & editing. AF: Data curation, Formal Analysis, Software, Writing – review & editing. AlP: Data curation, Formal Analysis, Software, Writing – review & editing. GL: Data curation, Formal Analysis, Software, Writing – review & editing. CM: Data curation, Formal Analysis, Software, Writing – review & editing. SL: Conceptualization, Data curation, Formal Analysis, Funding acquisition, Investigation, Methodology, Software, Writing – original draft, Writing – review & editing. GS: Conceptualization, Investigation, Supervision, Writing – original draft, Writing – review & editing. GV: Conceptualization, Formal Analysis, Funding acquisition, Investigation, Supervision, Writing – original draft, Writing – review & editing.
