# Supplementary material for: Cytokine dysregulation despite immunoglobulin replacement therapy in common variable immunodeficiency (CVID)
Source: Front Immunol. 2023 Sep 28;14:1257398. doi: 10.3389/fimmu.2023.1257398 (PMC10568625; doi:10.3389/fimmu.2023.1257398)
Supplement: Supplementary file 1 [file DataSheet_1.docx]

Supplementary Material

**Cytokine dysregulation despite immunoglobulin replacement therapy in Common Variable Immunodeficiency (CVID)**

**Remo Poto^1,3^, Antonio Pecoraro^1,3^, Anne Lise Ferrara^1,3^, Alessandra Punziano^1,3^, Gianluca Lagnese^1,3^, Carla Messuri^1,3^, Stefania Loffredo^1,2,3,4^, Giuseppe Spadaro^1,2,3^, and Gilda Varricchi^1,2,3,4^**

**sFig. 1.** Correlations between cytokines and IgG concentrations after IgRT in patients with INF-CVID_._

_
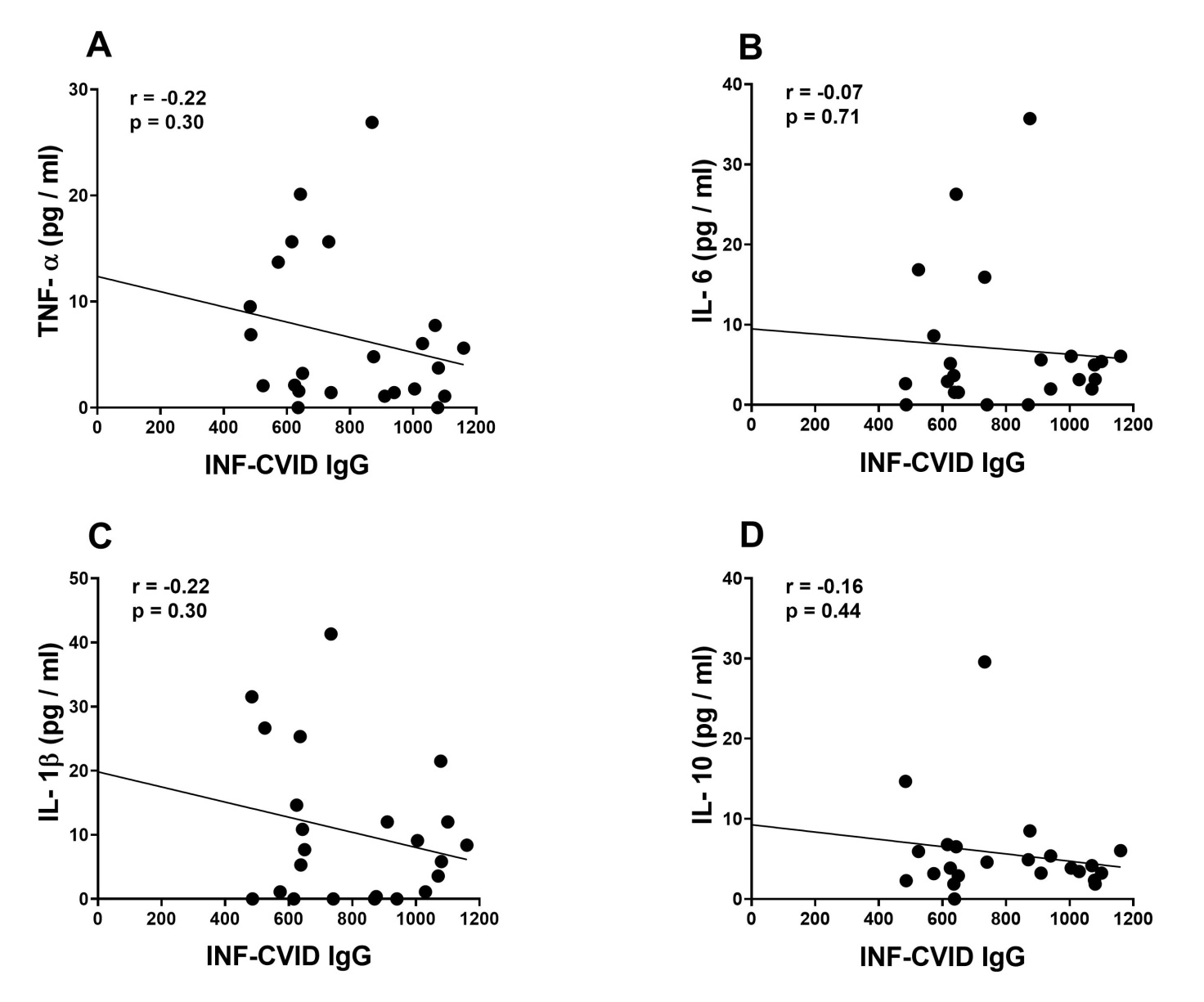
_

**sFig. 2.** Correlations between cytokines and IgG concentrations after IgRT in patients with NIC-CVID_._

**
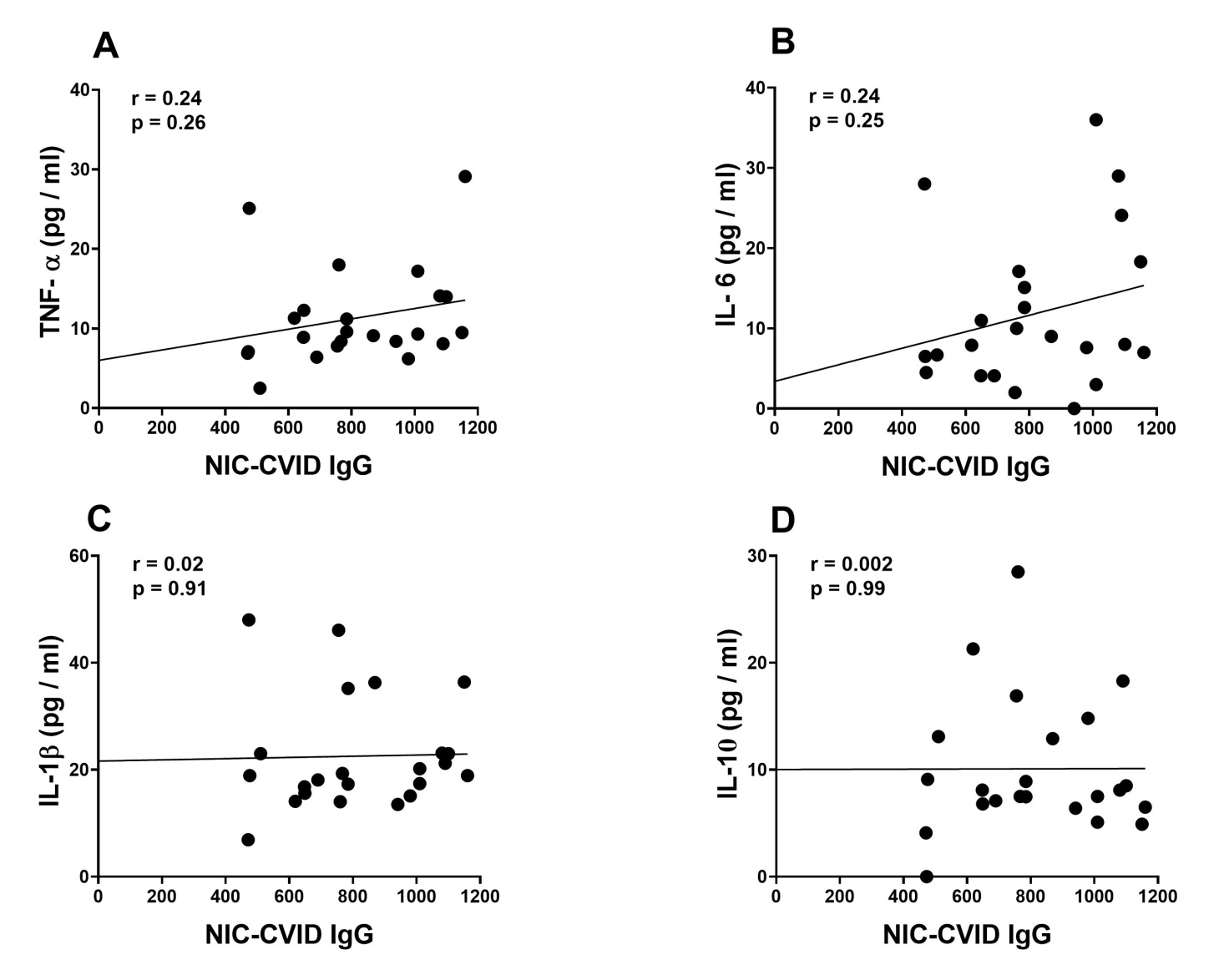
**

**sFig. 3.** Correlations between LPS and IgG concentrations after IgRT in patients with INF-CVID_._

_
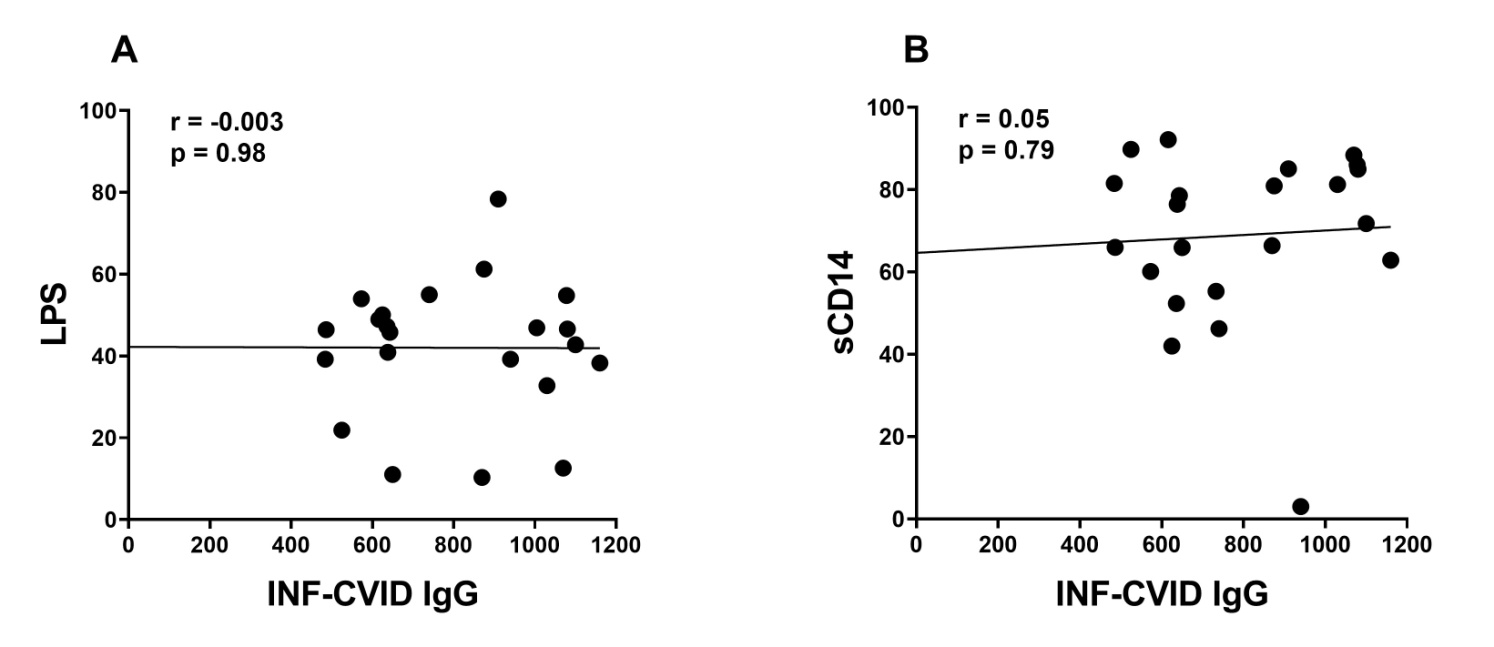
_

**sFig. 4.** Correlations between LPS and IgG concentrations after IgRT in patients with NIC-CVID_._

_
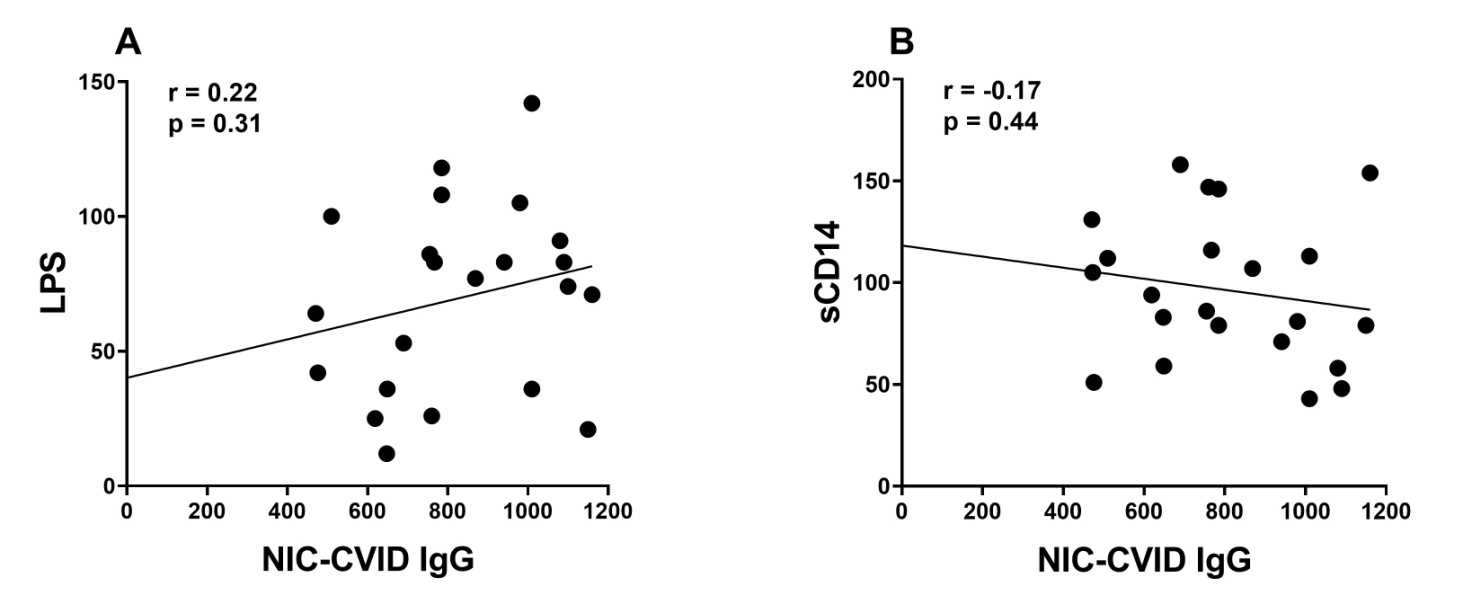
_

**sFig. 5.** Correlations between cytokines and sCD14 in patients with INF-CVID_._

**
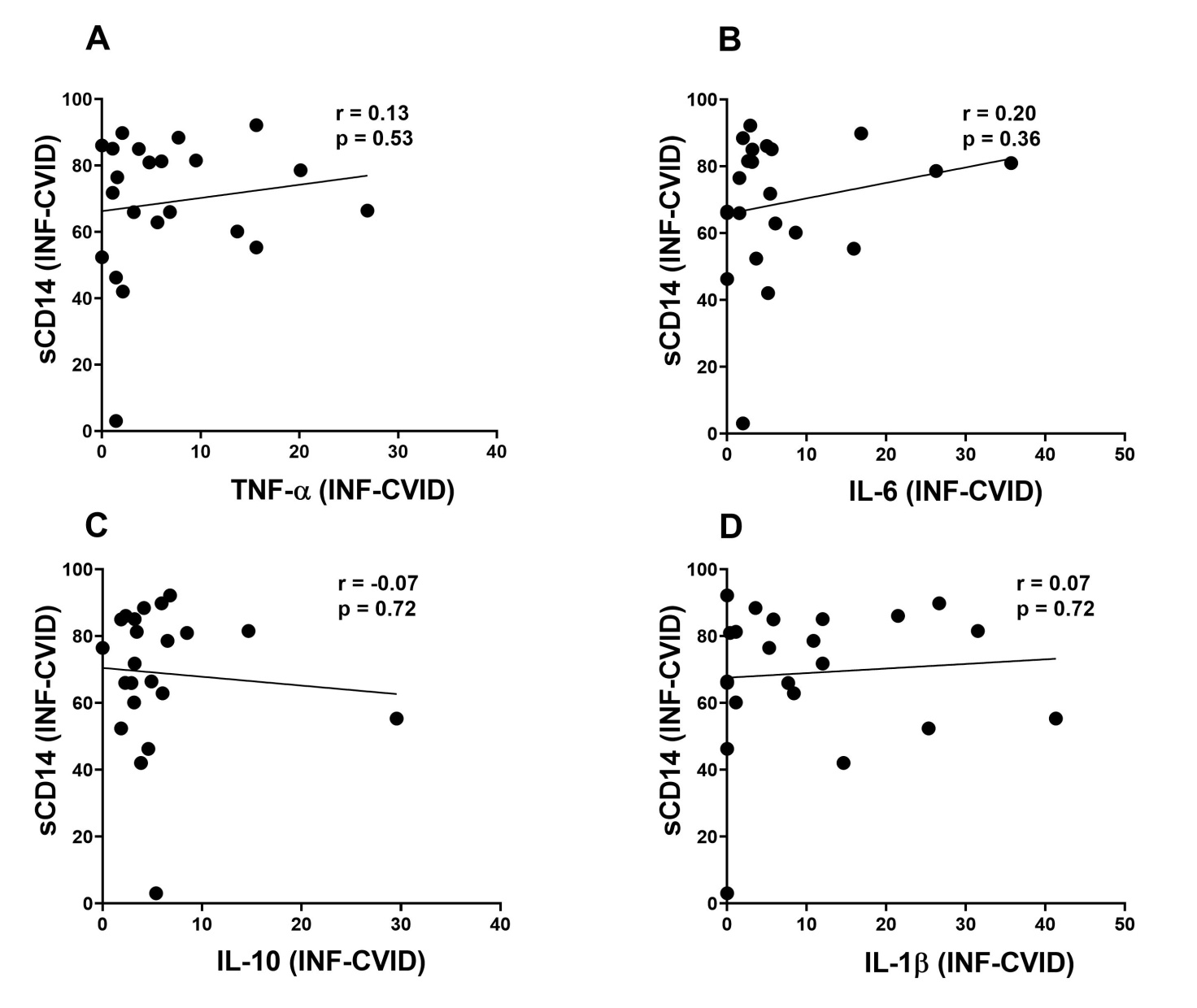
**

**sFig. 6.** Correlations between cytokines and sCD14 in patients with NIC-CVID_._

_
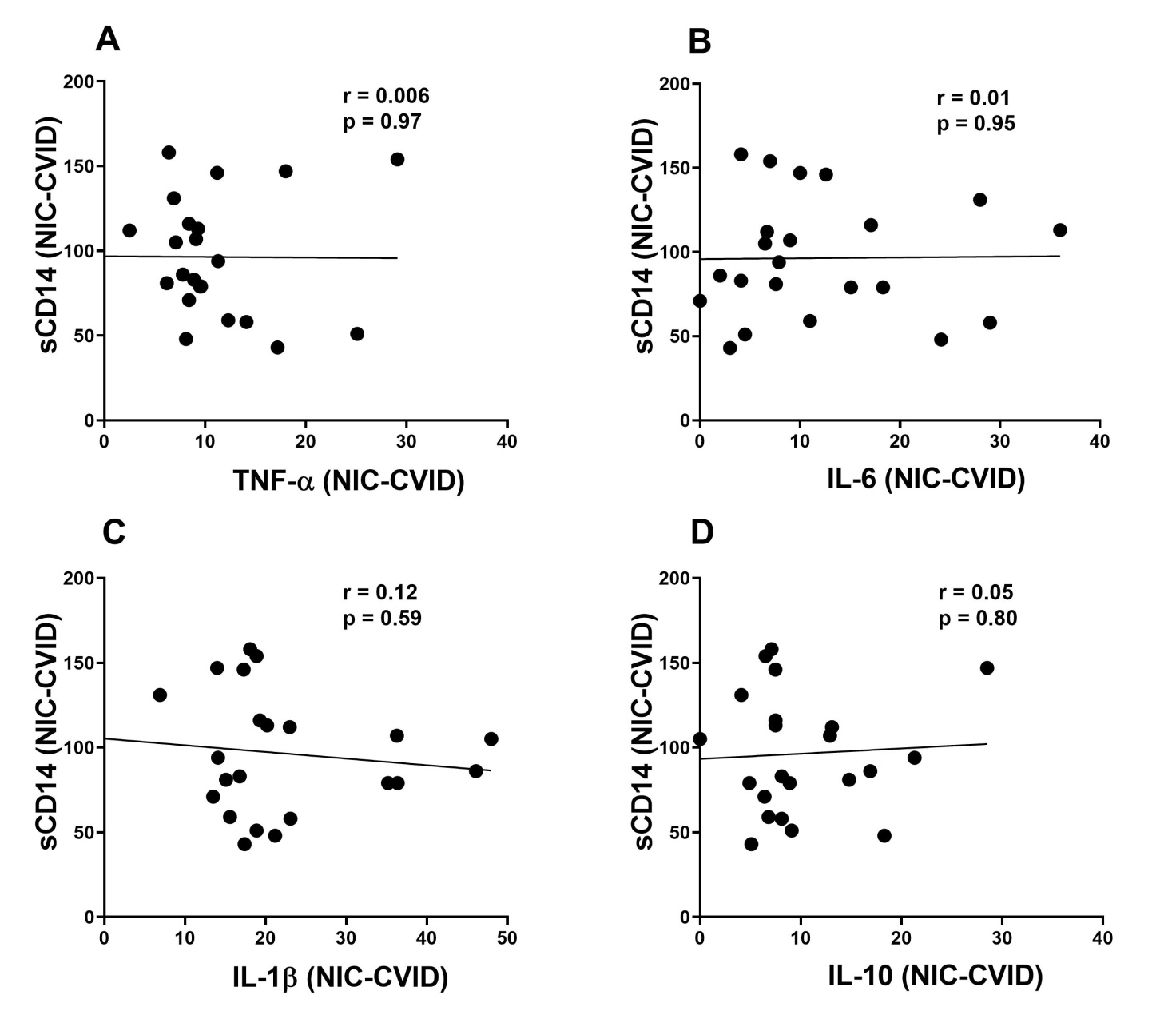
_

**sFig. 7.** Correlations between cytokines and LPS in patients with INF-CVID_._

**
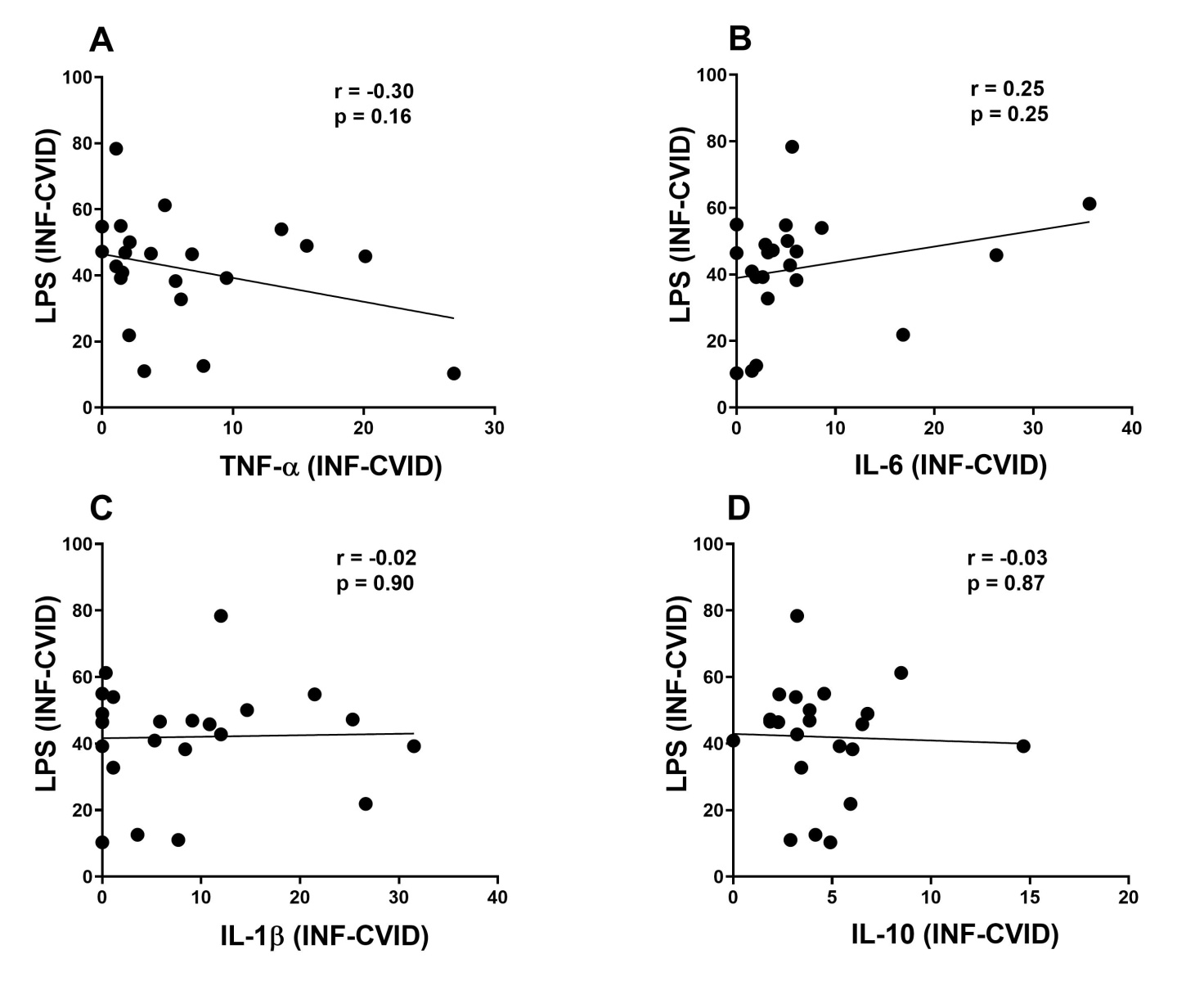
**

**sFig. 8.** Correlations between cytokines and LPS in patients with NIC-CVID_._

_
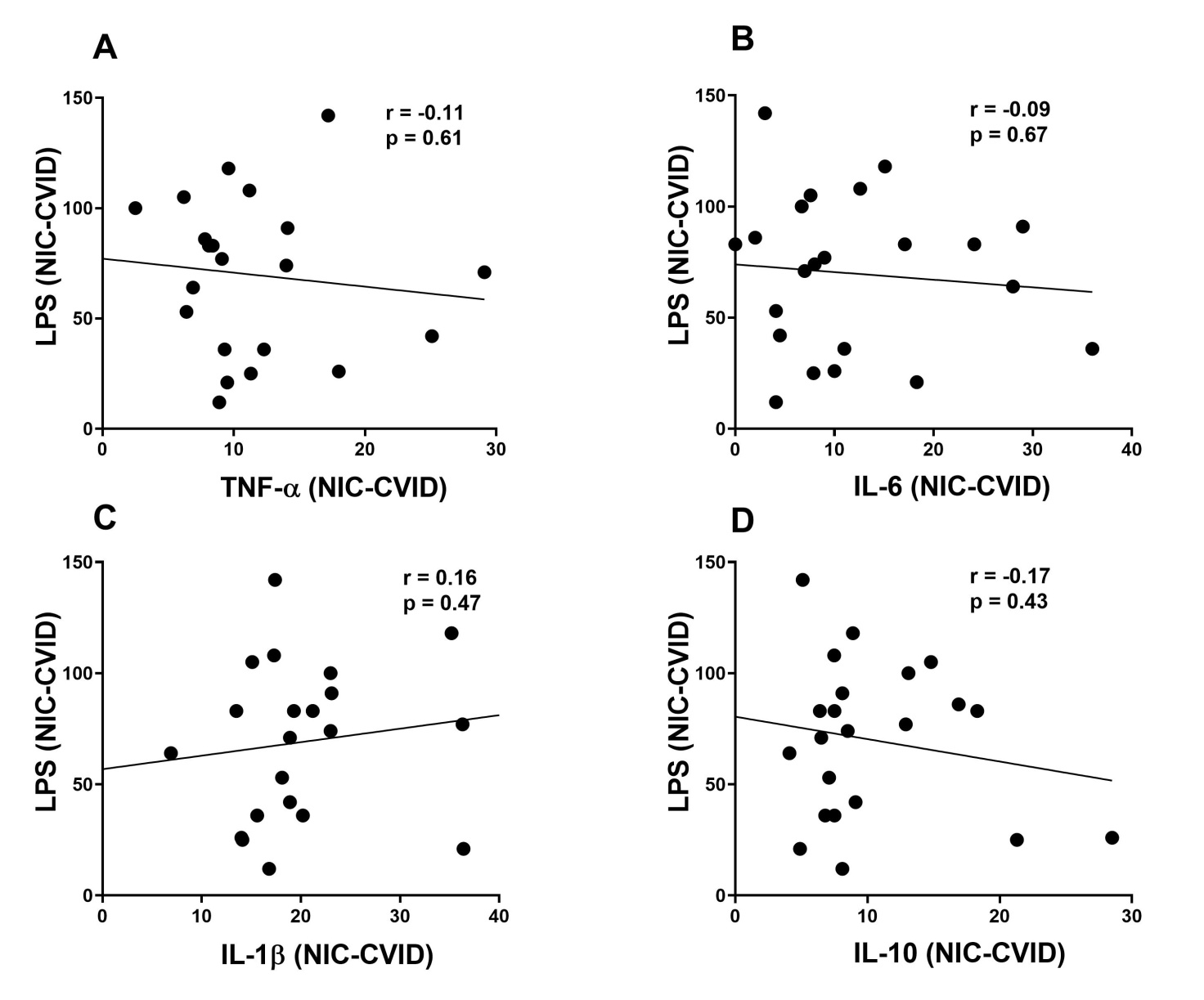
_

**sFig. 9.** Correlations between sCD14 and LPS concentrations in INF-CVID and NIC-CVID patients.

_
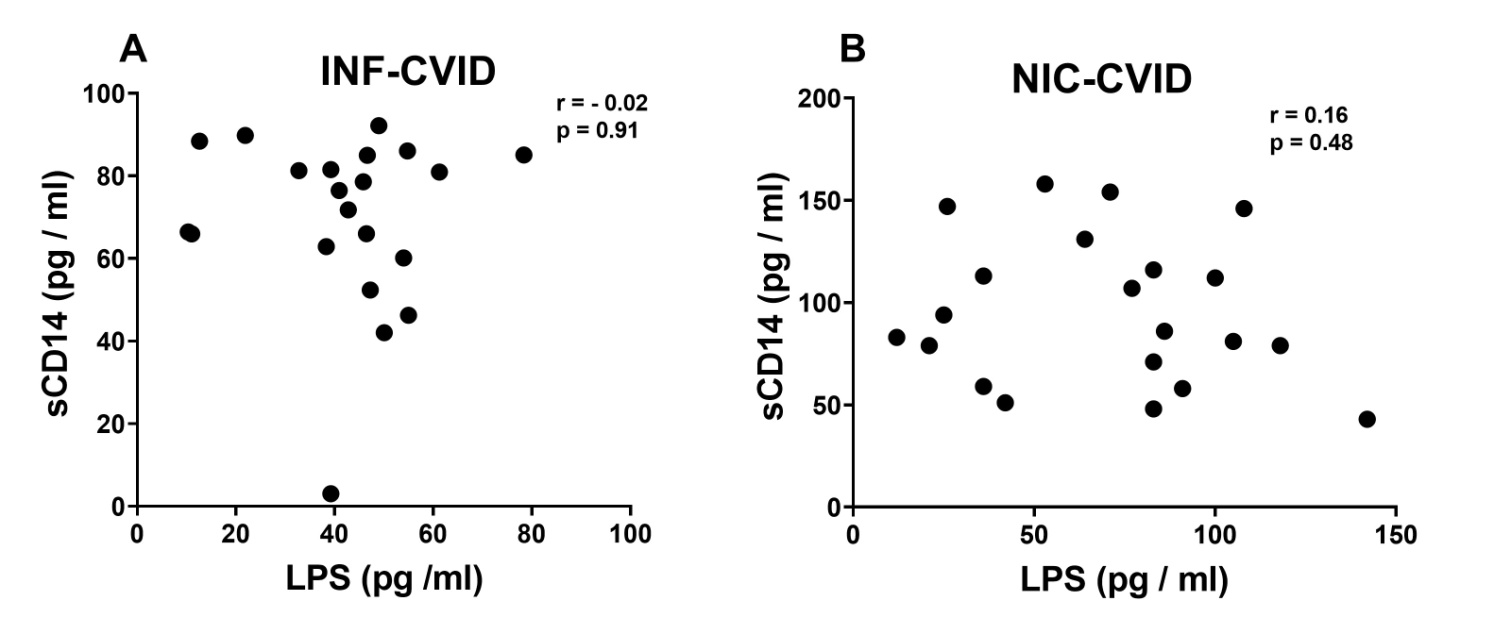
_

**sFig. 10.** Correlations between the different cytokines in the single subjects with INF-CVID_._

_
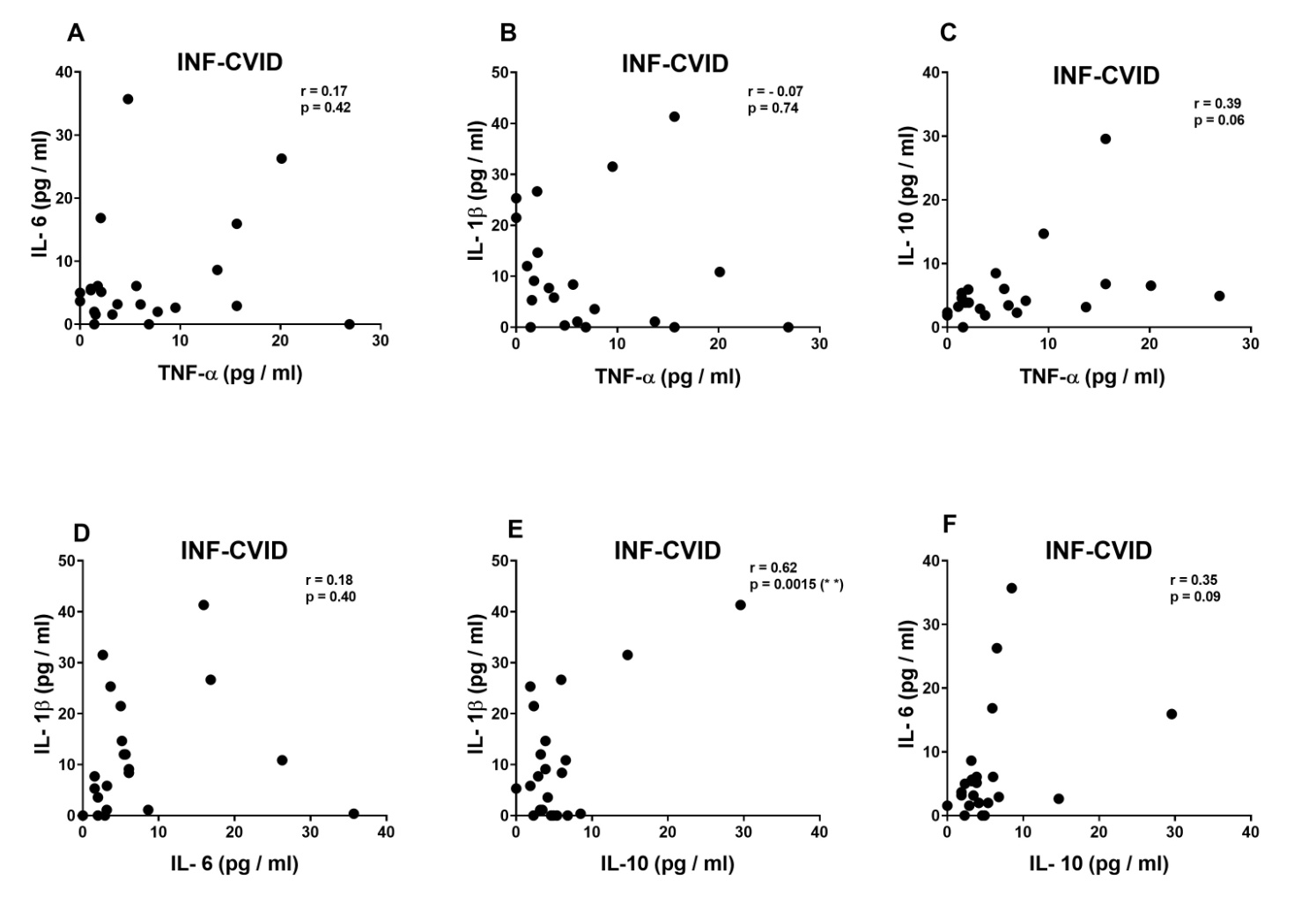
_

**sFig. 11.** Correlations between the different cytokines in the single subjects with NIC-CVID_._

_
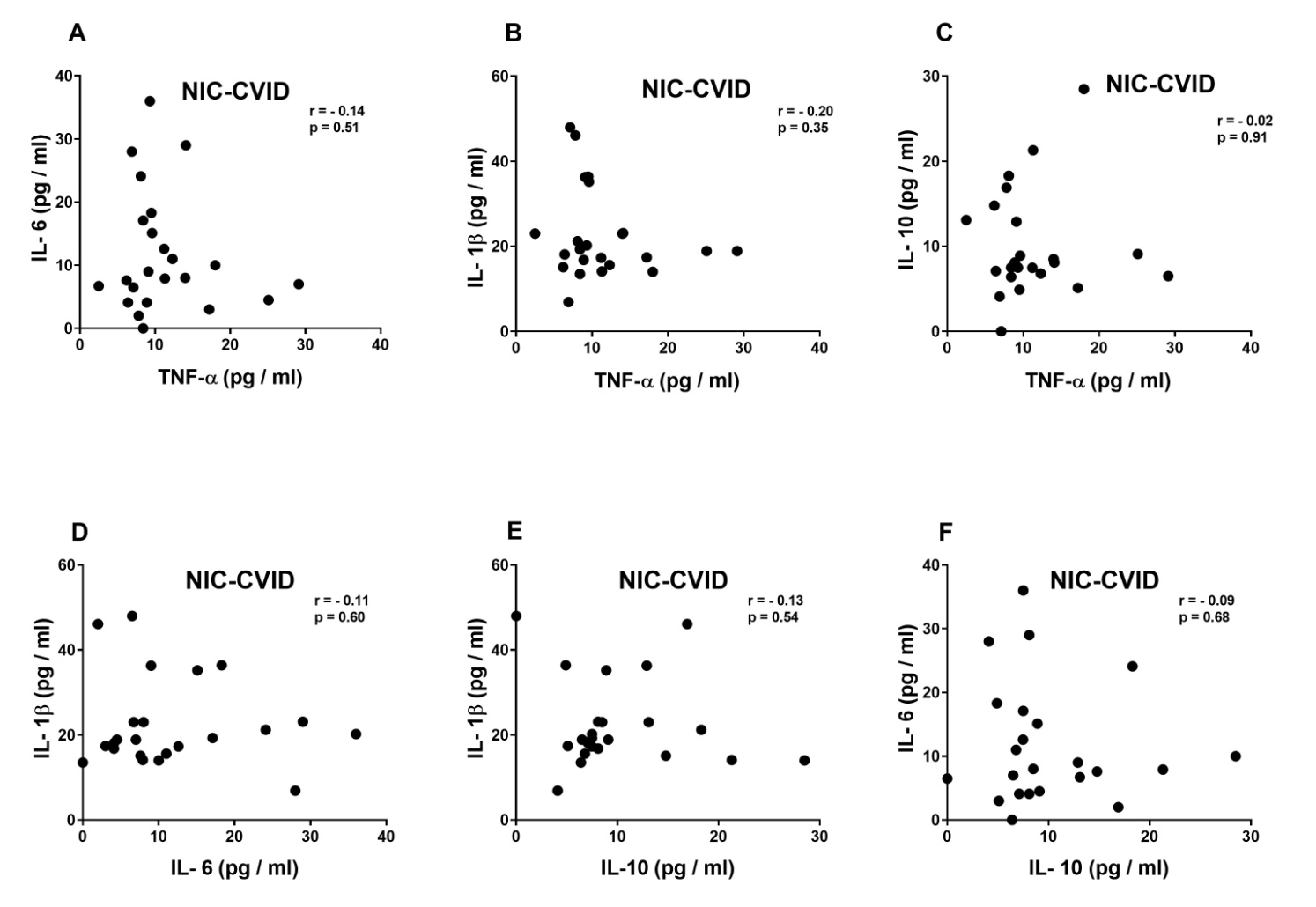
_

**
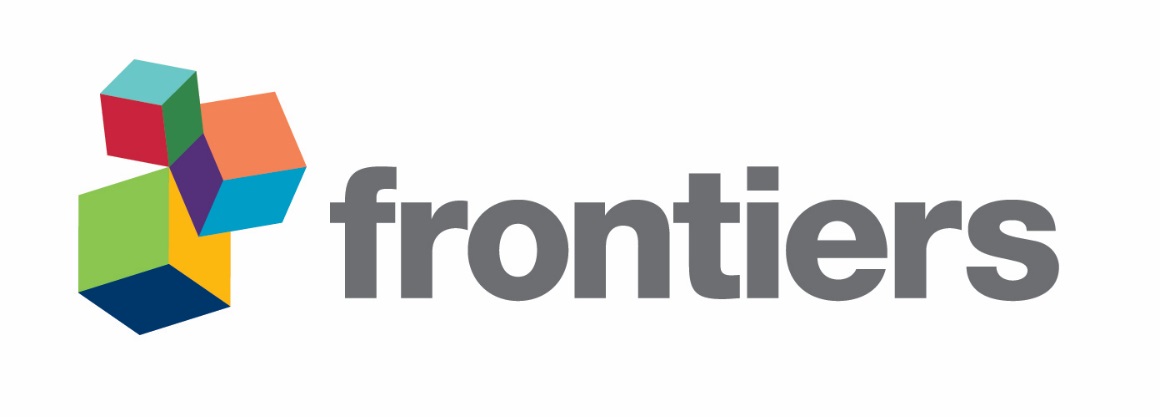
**
